# When “I” becomes “We”: ethical implications of emerging brain-to-brain interfacing technologies

**DOI:** 10.3389/fneng.2014.00004

**Published:** 2014-02-12

**Authors:** John B. Trimper, Paul Root Wolpe, Karen S. Rommelfanger

**Affiliations:** ^1^Department of Psychology, Emory UniversityAtlanta, GA, USA; ^2^Neuroethics Program, Center for Ethics, Emory UniversityAtlanta, GA, USA; ^3^Departments of Medicine and Pediatrics, Emory UniversityAtlanta, GA, USA; ^4^Department of Neurology, Emory UniversityAtlanta, GA, USA

**Keywords:** BTBI, brain-to-brain interfacing, ethics, emerging technology, responsibility, identity, neural privacy, neuroethics

## Introduction

The idea of creating a direct connection between a human brain and a computer has a long history in science fiction. The development of brain computer interfaces (BCI), technologies permitting direct communication between a user's brain and an external device, began to become a reality in the 1970s (Vidal, 1973), and have since captured the attention of scientists and the public alike. Initially conceptualized for military use—the initial work was funded by the National Science Foundation and the Defense Advanced Research Projects Agency (DARPA)—more recently BCIs have shown promise for therapeutic uses, providing hope for restorative and even enhanced human capacities.

Utilizing both invasive and non-invasive technologies, scientists are now capable of recording and translating activity from populations of neurons to operate external devices (e.g., O'Doherty et al., 2011). In early 2013, the technology took a leap forward as researchers replaced the external computer connection with a second embodied brain, dubbing the approach “brain-to-brain” interfacing (BTBI). The direct transfer of information between two brains raises new and important ethical issues. Below, we summarize the first two landmark studies in BTBI research, and then discuss ethical concerns relevant to BTBI as they are applied in clinical, research, and non-therapeutic domains.

## Key findings in BTBI

### Sensorimotor transmission between two rats

Two studies mark the first major advances in BTBI technology. In the laboratory of Miguel Nicolelis, Pais-Vieira et al. (2013) successfully transferred sensorimotor information from the brain of one rat into that of another. Rats were trained to complete a discrimination task in which illumination of an LED above one of two levers indicated which lever was rewarded. Rats were then grouped into either the “encoder” or “decoder” cohort and implanted with either recording or stimulating electrodes, respectively, in their primary motor cortices. The goal was to transfer task relevant motor information from encoder to decoder rats via electrical stimulation to investigate whether this transfer could allow the decoder to correctly perform the discrimination task in the absence of any other cues. With each correct decoder response, the encoder received an additional reward resulting in a feedback loop for encoders. Without BTBI, decoder performance was at chance; when BTBI was used, however, decoder performance was significantly enhanced, enabling animals to choose the correct lever when receiving neural information from encoders. The investigators found similar performance enhancements with the transfer of somatosensory information (i.e., discriminating aperture width). Pais-Vieira et al. (2013) also coupled the brains of a rat in North Carolina with a rat in Brazil, sending information via Internet, rather than to the adjacent cage. Here, too, decoder rats chose the correct lever more often when BTBI was implemented. A subsequent study by Deadwyler et al. (2013) demonstrated that cognitive information related to the encoding of a memory could also be transferred from one rat's brain to another's.

### Cross species neural interfacing

A second group, based in Harvard Medical School, utilized a non-invasive form of BTBI to transfer information from human to rat brains, marking the first interspecies brain interfacing study. Yoo et al. (2013) recorded signals from a human scalp and transformed these signals into tail-movement-evoking stimulation delivered to the brain of an anesthetized rat. Electroencephalography (EEG) was used to record neural activity from humans who were presented with a visual stimulus. When the human participant intentionally focused attention on the stimulus, a steady-state visual evoked potential (SSVEP), a robust electrophysiological signal corresponding to active visual focus, was produced. The SSVEP triggered a stimulation pulse delivered to an anesthetized rodent's scalp using focused ultra-sound (FUS), in which focused acoustic energy is delivered non-invasively to a targeted brain region (but see Davis and van Koningsbruggen, 2013 re: “non-invasive”). Delivery of the SSVEP-invoked FUS pulse reliably evoked rodent tail movement. Yoo et al. (2013) suggest that interspecies BTBI may eventually be used in the reverse direction, from non-human animals to humans, for such things as enhancing our sensory systems (e.g., improving olfaction by linking our olfactory systems to those of a dog) or aiding in search-and-rescue operations, linking our brains with those of the search-and-rescue animal.

## Ethical implications

The very act of linking two brains together to transfer information raises a variety of ethical and safety concerns. Though born of two approaches, extraction and delivery of information, that are each privy to their own suite of ethical issues, BTBIs are a novel means of information transfer which bypasses the customary sensory means for the brain to apprehend information from another individual. Table 1 summarizes ethical issues associated with each aspect of BTBI technology. Such a novel approach demands careful consideration, especially as we develop human-to-animal and human-to-human BTBI technologies. Here we will discuss three categories of concern: potential violations of privacy, human enhancement, and agency and identity.

**Table 1:** **Ethical concern by component of brain-to-brain interfacing**.

**Extraction of information**	**Delivery of information**	**Combination**
**Privacy**	**Potential for adverse sequelae**	**Responsibility for damages incurred while operating BTBI**
E.g., safely storing and protecting the wealth of data extracted via neural recording techniques (Alpert, 2007)	Invasive and non-invasive stimulation techniques lack a proper body of research into potential negative long term effects (Bell et al., 2009; Horvath et al., 2014)	Some precedence with BCI (Tamburrini, 2009), but linking two brains adds several complications
**Informed consent**	**Informed consent**	**Ownership of BTBI generated ideas**
E.g., modifying hardware/software parameters to optimize recording in a non-responsive patient (Vlek et al., 2012)	E.g., keeping patient informed about reasonable expectations and potential risks associated with brain stimulation (Rabins et al., 2009)	If ideas are generated during the use of BTBI, who rightfully owns those ideas?
	**Enhancement**	**Agency**
	E.g., stimulation being used to enhance learning and memory (Bass et al., 2012)	Issues regarding the inability/ability to control one-self/another being while operating BTBI devices
	**Coercion**	**Personhood**
	Possibility of eventual non-consensual use to control or coerce thought or behavior	How does one define oneself as a person when brains are wired between individuals?

*BTBI can be broken down into two components: extracting neural information and delivering neural information. Each of these components is associated with several of its own ethical concerns. However, when combined and manifested as BTBI, several novel ethical considerations emerge*.

### Neural privacy

Similar to any technology that can extract meaningful information from an individual's brain/body (e.g., fMRI, genetics, EEG), BTBI raises concerns of privacy. While considerable effort has been made to protect genetic information, few safeguards have been created for neural data. Priorities in mapping the human connectome, as with the new US Brain Initiative and the EU's Human Brain Project, foreground the potential for brain-based data to be as powerful and identifiable as genetic data. In addition, fMRI technologies are already being used to reconstruct videos observed by participants (Nishimoto et al., 2011), decode the category of objects viewed by participants (Haxby et al., 2001), and decipher whether or not subjects are lying (Davatzikos et al., 2005). BTBIs add another dimension to the neural privacy concern; not only is information extracted and decoded from the transmitting brain, it is introduced to a receiving brain, presumably without the ability of that brain to refuse or inhibit the impulse. The specter of introducing various kinds of information coercively is also a plausible ethical concern. Because BTBI-related neural information can be transferred over the Internet (as in Pais-Vieira et al., 2013, above), conceivably, one could “hack” another's neural device as has been done with heart pacemakers (Halperin et al., 2008). In addition, these transmissions could be communicated discretely, with items such as “epidermal electronics” (Kim et al., 2011)—extremely thin grids of electrical sensors applied directly to the outside of the skin, similar to a temporary tattoo—placed on the forehead under hair, for instance. BTBI connections, especially through wireless transmissions, could eventually allow soldiers or police—or criminals—to communicate silently and covertly during operations.

Another concern, though still far in the future, is the eventual possibility to use this technology coercively. If thoughts can be planted, or behavior compelled, through interfaces that send stimulation or information directly to the brain, it is theoretically possible at some point that such technology might be used without consent to control the behaviors of prisoners, for example. While the current state of the technology is too primitive for such use now, vigilance is imperative as this research continues.

### Enhancement

As is the case with other neural interventions, such as transcranial direct current stimulation (TDCS) (Coffman et al., 2013) and pharmacological substances (Greely et al., 2008), BTBI has the potential to be used to enhance human cognition (Bostrom and Sandberg, 2009) or well being (Earp et al., 2014). For example, coupling brains could one day provide an advantage to students, enhancing speed of knowledge or skill acquisition. BTBI facilitated learning could widen the gap of social inequities in education and other areas even further, as it is likely that very few will be able to afford initial prototypes.

Though progress in brain interfacing technology must first overcome several technological obstacles (Lebedev and Nicolelis, 2006), the future promises advances that will encourage individuals to experiment with these technologies for enhancement purposes, perhaps even creating home-made brain interfacing devices as has already been done with TDCS (Fitz and Reiner, 2013) despite substantial concern regarding the readiness of TCDS technology (Horvath et al., 2014). Do-it-yourself kits for creating TDCS-like devices are already pre-selling from some websites (www.foc.us) and instructional videos for home-made TDCS devices can be found on YouTube. Using EEG, Presacco et al. (2012) recently demonstrated success decoding detailed information about limb movements recorded as participants walked on a treadmill. Likewise, Choi (2013) decoded with high accuracy the shoulder and arm joint movements of participants based on non-invasive recordings. If this information could be transferred via stimulation to the relevant regions of a second individual's (or non-human animal's) brain, it may be possible to control the limbs of the receiving subject via encoder's neural activity. For example, a recent article (Sakurada et al., 2013) reports experiments in which EEG was used to control a Brain-Machine Interface (BMI) suit worn by the user, allowing the suit to grasp a ball and subsequently drop the ball in a target location.

Some have argued that such use of BCIs outside the context of therapeutics violate individual authenticity, disrespects the “limits of nature,” and puts us as risk of losing “what makes us human” (Kass, 2003; Demetriades et al., 2010). More nuanced concerns include issues like the loss of autonomy and the potential for coercive control over another creature. Clearly the implications of these technologies for changing the nature of human functioning and capacity deserve careful thought and scrutiny as the technologies develop.

### Agency and identity

If one is defined by his or her neurophysiology, a neuro-essentialist view (Roskies, 2002; Racine et al., 2005), then how is individuality defined when a brain is synched with another's, or perhaps many others'? Might one's concept of the self yield to a new, communal sense of identity? Who owns thoughts generated in brain-to-brain interfacing? Related to questions of identity are concerns over the issue of agency and responsibility. Tamburrini (2009), for example, points out that by using BCI technology, users accept some degree of responsibility for actions of the machine with which s/he is integrated. Therefore, s/he may be at fault for damage (such as a BCI wheelchair damaging someone's property). In the case of BTBI, on the other hand, where it may not be clear that a “decoder” is acting on her own accord, the assessment of responsibility becomes more complex.

The US and UK defense departments are significant funders of BMI research for cognitive and performance enhancement (Hoag, 2003; The Royal Society Science and Policy Centre, 2012). With its liberal approach to “enhancement” (e.g., Caldwell and Caldwell, 2005) and history of researching BMIs for wounded soldiers (Kotchetkov et al., 2010), the military may be the first to employ BTBI in humans. Yet BTBI in combat may make issues of responsibility even more confusing. Imagine that a soldier in ground combat with a helmet mounted camera is able to neurally receive information directly from a second soldier monitoring the video in real-time. If the monitoring individual detects a threat on the video, this information could rapidly be transferred to the ground soldier who could respond with greater speed and potentially, greater accuracy. While benefits, such as improved reaction time, can readily be identified in such an example, questions arise when one considers potential accidents, such as injury by friendly fire or collateral damage. Who would be responsible for the soldier or civilian deaths—ground or monitoring soldier? What if the stimulation pattern was misinterpreted by the ground soldier or computer's algorithm, or intentionally transmitted by the monitoring individual? While current BTBI research is focused on transferring information, future work might include transfers emotion or even false memories, as was done recently in mice (Ramirez et al., 2013).

Pilots operating drone planes miles from combat zones sometimes report experiencing PTSD (Otto and Webber, 2013). Some speculate that such symptoms are similar to “vicarious traumatization,” found in mental-health workers who have listened to first-hand accounts of war-time violence (Reardon, 2013). Arguably the mental impact of technologies that remove the actor one-step from the action may warrant a new approach by psychiatry (Wolpe, 2007). Thus, personal ethical dilemmas and potential mental health challenges that may arise from using such interfaces require further consideration.

## Conclusion

Recent advances in brain interfacing technologies now allow for direct communication between two individuals' brains (Pais-Vieira et al., 2013; Yoo et al., 2013). To date there is no legislation regulating informed consent and protecting personal data extracted via BCIs, much less BTBIs, either therapeutically or outside of the clinical and research context. Further, no formal protocols are in place for how to conduct research using these technologies, with humans or non-human animals. These studies continue to advance; recent unpublished preliminary data by researchers at University of Washington mark the first human to human BTBI, utilizing non-invasive EEGs and transcranial magnetic stimulation. Further the non-invasive nature of these technologies, the ease of engineering the relevant hardware, and the enthusiastic DIY culture interested in cognitive enhancement make exploring these ethical issues especially pressing. Ethical discourse around BTBI must keep pace with the advances in technology in order to prepare for possible life-changing implications of BTBI use. We have seen the public uproar and subsequent opposition to technology, as with mammalian cloning with Dolly, when there is a failure to prepare the public for such innovation (Wolpe, 2006). In order to best prepare the public to understand the implications of BTBI, ethicists and scientists must work together to ensure that the technology is developed with the highest ethical standards.

## Author contributions

John B. Trimper and Karen S. Rommelfanger made equal contributions to the conceptualization and writing of the present manuscript. Paul Root Wolpe made significant contributions to writing of the present manuscript.
